# The Efficacy and Safety of Zaoren Anshen Capsule in Combination with Zolpidem for Insomnia: A Multicentre, Randomized, Double-Blinded, Placebo-Controlled Trial

**DOI:** 10.1155/2022/5867523

**Published:** 2022-11-23

**Authors:** Xiangzhen Zhu, Ming Tao, Haoyu Hu, Jingfang Gao, Jiong Chen, Tiaotiao Lu, Xiaole Wang, Wei Kong, Lijun Lv, Minjun Wei

**Affiliations:** ^1^The First Affiliated Hospital of Zhejiang Chinese Medical University (Zhejiang Provincial Hospital of Traditional Chinese Medicine), Hangzhou, China; ^2^Zhejiang Chinese Medical University, Hangzhou, China; ^3^Jinhua Hospital of Traditional Chinese Medicine, Jinhua, Zhejiang, China; ^4^Tongde Hospital of Zhejiang Province, Hangzhou, China; ^5^The Second Affiliated Hospital of Zhejiang Chinese Medical University (Xinhua Hospital of Zhejiang Province), Hangzhou, China

## Abstract

**Purpose:**

Insomnia is the most common sleep disorder with high rate of prevalence, persistence, and leads to negative consequences. The mainstays of insomnia treatment have limitations due to either the side effects of hypnotics or limited accessibility to cognitive behavioral therapy. This study aims to compare the efficacy and safety of the traditional Chinese medicine (TCM) Zaoren Anshen capsule alone or as an adjunct treatment with different doses of the nonbenzodiazepine medication zolpidem tartrate in treating insomnia.

**Method:**

This randomized, double-blind, multicentre placebo control trial was conducted in 131 patients with chronic insomnia. The patients were randomly assigned to one of the following four regimen groups: Group ZA + *Z*5 : Zaoren Anshen capsule and 5 mg zolpidem tartrate (*n* = 32); Group Z5: 5 mg zolpidem tartrate and placebo capsule (*n* = 35); Group Z10 : 10 mg zolpidem tartrate and placebo capsule (*n* = 32); Group ZA : Zaoren Anshen capsule and placebo pill (*n* = 32). The drugs were administered for 4 weeks. All patients were evaluated by the Insomnia Severity Index (ISI) at 0, 2, 4, 5, and 6 weeks, and adverse events were recorded.

**Result:**

There are significant differences in the comparison between the four groups at each treatment stage (*P* < 0.05). Repeated measurement analysis of variance (ANOVA) of ISI scores in each treatment stage of the four groups exhibits significant differences in time effect, intergroup effect, and interaction effect (*P* < 0.05). After four weeks of drug administration, the treatment efficacy is similar in Groups ZA + *Z*5 and Z10 (93%) and in Groups Z5 and ZA (62% and 65%, respectively). Groups ZA + *Z*5 and Z10 present significantly lower ISI scores compared with Groups Z5 and ZA (*P* < 0.05), which indicates better treatment response of Groups ZA + *Z*5 and Z10. No significant difference was observed in the incidence of adverse events between the groups.

**Conclusion:**

Zaoren Anshen capsule can effectively treat insomnia disorder either alone or in combination with zolpidem tartrate. A preferred combination of TCM Zaoren Anshen capsule with zolpidem can provide a magnified therapeutic efficacy with fewer side effects than zolpidem-only management, clinical trial registration number: Chinese Clinical Trial Registry ChiCTR-IPR-1600969.

## 1. Introduction

Insomnia is the most common sleep disorder with an estimated prevalence of 10–20% in the general population [1]. The occurrence of insomnia increases with age. It is reported that 30%–48% of the elderly population has ever experienced symptoms related to insomnia, which can be partially attributed to developmental changes in sleep architecture [2].

Besides being prevalent, insomnia is also highly persistent and recurrent. The persistence rate of insomnia over a 5-year duration can reach up to 40%. Patients with insomnia exhibit high risk of physical and mental illness such as cardiovascular disease [3, 4], diabetes [5–7], and chronic kidney disease [8, 9]. Insomnia is also associated with chronic pain [3, 10], dementia [11], anxiety [12, 13], and depression [14–16]. All of the conditions will further impair the quality of life and increase morbidity and mortality [17, 18]. Impaired attention and awareness secondary to poor sleep have been reported to significantly increase accidents and injuries [19, 20]. The distress associated with insomnia is now a public health concern. Thus far, tremendous direct and indirect costs associated with insomnia impose a heavy financial burden on both individuals and society [21–23]. Initiating safe and effective treatment in a timely manner is appreciated because it can not only reduce the functioning disability caused directly by insomnia, but also prevent the associated negative physical and psychological consequences.

Current mainstay of insomnia treatment includes both pharmacological and nonpharmacological approaches [24–26]. However, in clinical practice, both approaches have limitations. Drugs commonly used to treat insomnia such as benzodiazepine receptor agonists cause adverse effects such as hangovers, dizziness, and fatigue [27–29]. The long-term use of these drugs may lead to drug dependence as well as withdrawal when discontinued inappropriately [30]. Due to the limitations and side effects, hypnotic medication should be used under the care of a physician for short periods. When it comes to the nonpharmacological approach, cognitive behavioral therapy for insomnia (CBT-I) has been proven to be effective and recommended as the first-line treatment for insomnia. Studies have shown that CBT-I exhibits short-term effects similar to those of medication, whereas the therapeutic gains could be sustained for a longer time period with CBT-I [31, 32]. However, there are various conditions limiting patients from receiving efficacious CBT-I treatment, such as poor geographical accessibility to healthcare, lack of trained therapists, and relatively high cost [32, 33].

Due to the side effects of hypnotics and limited accessibility to CBT-I, alternative approaches such as traditional Chinese medicine (TCM) have become viable complementary strategies [34, 35]. TCM exhibits a long history in Chinese medical practice and is progressively being recognized in the world. Systematic analyses of randomized controlled studies on TCM for insomnia have demonstrated the safety and efficacy in improving sleep quality [36–38]. Zaoren Anshen capsule is a Chinese patent medication that has been made in accordance with the standards of a nonbenzodiazepine sedative hypnotic to treat insomnia. The capsule comprises three TCM ingredients, namely Suan Zao Ren (Ziziphi Spinosae Semen, 417 mg/capsule), Wu Wei Zi (Schisandrae Chinensis Fructus, 70 mg/capsule), and Dan Shen (Salviae Miltiorrhizae Radix et Rhizoma, 167 mg/capsule) [39]. Clinical studies have suggested that Zaoren Anshen capsule has higher efficacy than that of placebo and that it produces a similar effect as benzodiazepines with fewer side effects [40, 41]. Unfortunately, these studies have methodological limitations such as lack of an active control group, small sample size, and lack of long-termfollow-up. In addition, it remains unclear whether the combination of TCM with drugs can provide additional therapeutic gains. Thus, a study with a robust design is required to evaluate TCM Zaoren Anshen capsule's efficacy in treating insomnia.

This study aims to compare the efficacy and safety of the TCM Zaoren Anshen capsule alone or as an adjunct treatment with different doses of the nonbenzodiazepine medication zolpidem tartrate in treating insomnia.

## 2. Methods

### 2.1. Study Design

The present randomized, double-blind, and parallel placebo-controlled study was conducted in 135 participants recruited from four hospitals (Zhejiang Provincial Hospital of Traditional Chinese Medicine (*n* = 50), Xinhua Hospital in Zhejiang Province (*n* = 41), Tongde Hospital in Zhejiang Province (*n* = 20), and Jinhua Hospital of Traditional Chinese Medicine (*n* = 24)) in Zhejiang Province of China from December 2016 to December 2018. After baseline assessment, the participants received a 4-week intervention, requiring assessment during weeks 2 and 4, and reassessment at weeks 5 and 6. The study was approved by the Ethics Committee of Zhejiang Provincial Hospital of Traditional Chinese Medicine (number: 2016-KL-009-01) and registered at the Chinese clinical trial registry (registration number: ChiCTR-IPR-16009669).

### 2.2. Randomization and Blinding

The participants were blinded to the group allocation. The random coding table was established by the clinical research data management and statistical unit. The blinding code was sealed and submitted to the responsible clinical research unit and sponsor for proper preservation. A statistical professional who did not have contact with the participants during the study period conducted the randomization procedure. The entire drug coding process was documented by a person unrelated to the present clinical study. The assessors who were responsible for drug prescription and assessments were also blinded to the group allocation. The randomization list was kept confidential throughout the study period unless any adverse event was reported.

### 2.3. Participants

The participants were recruited from four hospitals in Zhejiang. These participants' age varied from 18 to 65, educated beyond grade 3, and diagnosed with insomnia disorder according to the Diagnostic and Statistical Manual of Mental Disorders version 5, and with a Pittsburgh Sleep Quality Index (PSQI) score >7. Participants with following conditions were excluded from the study: ① patients who were professional drivers or aerial workers; ② patients who were already on sleep medications; ③ pregnant and lactating women; ④ patients with severe chronic diseases such as cancer, cardiovascular diseases, and kidney dysfunction; ⑤ those allergic to zolpidem tartrate or Zaoren Anshen capsules; ⑥ patients with mental disorders or with severe epilepsy; ⑦ patients with psychiatric disorders such as depression (Hamilton Depression Rating Scale-17 (HAMD) ≥ 17), anxiety (Hamilton Anxiety Rating Scale (HAMA) ≥ 14), and alcohol or drug abuser; ⑧ those who had participated in other drug clinical trials within 3 months of enrollment; and ⑨ those who were unable to provide informed consent were excluded from the study.

### 2.4. Interventions

Eligible participants were randomly assigned to one of the following four groups: Group ZA + *Z*5 : Zaoren Anshen capsule and 5 mg zolpidem tartrate; Group Z5: placebo capsule and 5 mg zolpidem tartrate; Group Z10 : Placebo capsule and 10 mg zolpidem; and Group ZA : Zaoren Anshen capsule and placebo pill.

After randomization, the participants were administered the therapy as per their assigned regimen once a day before bedtime for 4 weeks. After 4 weeks, the drug was discontinued and the withdrawal reaction was observed. The participants were provided with health education during the treatment.

During the trial, drugs other than those specified in the dosing regimen such as other TCMs, Western medicine for insomnia, and external treatment methods were not used as these drugs may affect drug efficacy evaluation. All the participants had to comply with the combined medication regulations during the period from drug initiation to the assessment of efficacy and safety.

The blinding code could be broken urgently when an emergency occurred during the study (such as a serious adverse event), or when a patient without any former treatment recorded need to be rescuredin the study. Emergency blindness breakingmust be decided by the main investigators. The cause, time, and place of breaking the code need to be recorded in detail and signed.

### 2.5. Efficacy Evaluation

Insomnia symptoms were measured using the Insomnia Severity Index (ISI). ISI is a 7-itemself-reported questionnaire that assesses the insomnia severity over 2 weeks. The treatment efficacy was measured by changes in the pre- and postintervention ISI score.

ISI decline rate = (pre-post ISI score difference)/pre-ISI score × 100%.

The clinical significance and parameters of the treatment were defined as follows: ① recovery: decline rate ≥95%; ② significant effect: decline rate is 60%–94%; ③ effective: decline rate is 30%–59%; and ④ no change: decline rate <30%. Effective rate = (the number of cured cases + the number of significant cases + the number of effective cases)/the total number of cases in this group × 100%.

#### 2.5.1. Adverse Events

Adverse events reported by patients during the intervention and follow-up period were recorded using an adverse event form. A description of the adverse events, time duration, severity, seriousness, adverse event outcome, and relationship with the experimental procedure were recorded. The number of adverse events was used to assess the safety of experimental drugs.

### 2.6. Statistical Analysis

All statistical tests were two-sided, and *P* values ≤ 0.05 were considered statistically significant. Statistical analysis was performed using SPSS 22.0. Continuous data were analyzed with ANOVA and repeated measurement ANOVA. Mean, standard deviation, effect size, and the *P* value were recorded. The pairwise *t*-test was used to compare between the pre- and posttreatment changes in the groups and baseline values in the screening period. The data that did not meet the normal distribution were represented by median (upper quartile and lower quartile). Rank-sum tests were used for comparison between the groups. Counting data of each visit in different treatment groups were described statistically by frequency (composition ratio). Pre- and posttreatment changes between the two groups were analyzed using the *χ*2 test or nonparametric test.

#### 2.6.1. Full Analysis Set

The full analysis set (FAS) refers to the ideal subject set as close as possible to the intent analysis principle (main analysis should include all randomized subjects). The data set was eliminated in the smallest and most reasonable method from all randomized subjects. The missing ISI, PSQI, HAMD, and HAMA values were estimated by carrying forward the last observation data carry-over method (LOCF) to the place where the study data were missing. The result was consistent with those in the beginning of the study. The FAS population was used for the analysis of primary and partial secondary efficacy indicators.

#### 2.6.2. Per Protocol Set

Patients with drug dosage of 80%–120% exhibited superior compliance, did not use prohibited drugs during the study period, withdrew the drug early for 2 weeks without any change or exacerbation, and completed the CRF regulations. The per protocol set (PPS) population was used for analyzing primary and secondary efficacy indicators.

#### 2.6.3. Safety Set (SS)

All patients using at least one study drug with at least one safety evaluation record constituted the safety analysis population in the present study. Safety population is the main population for safety evaluation in this study.

## 3. Result

### 3.1. Enrollment and Completion

**Figure 1:**
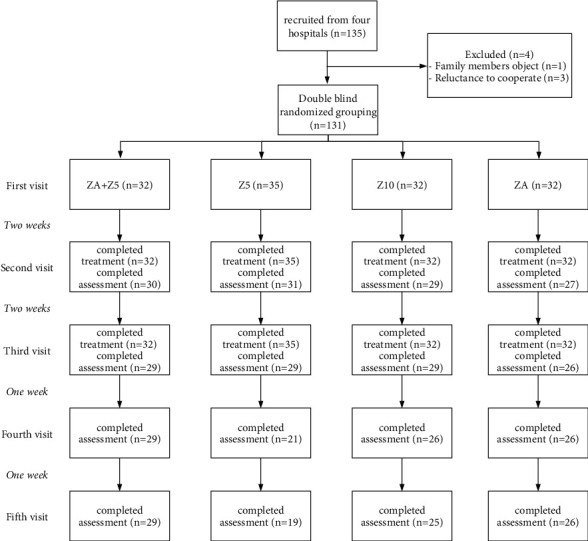
Flowchart of recruitment and randomization processes.

All the participants from Groups ZA + *Z*5 and Z5 completed the study, whereas one patient from Group Z10 and three patients from Group ZA dropped out of the study. The overall shedding rate was 2.96%.  Figure 1 shows the recruiting and randomization processes.

### 3.2. Participant Characteristics

Participant characteristics are presented in Table 1. The four groups did not differ in terms of age, gender, height, and weight (*P* > 0.05).

### 3.3. Clinical Efficacy

ISI scores of the four groups at each treatment stage are presented in Table 2. No significant difference was observed in the ISI scores between the groups in baseline (*P* > 0.05). However, a significant difference was observed between the groups during weeks 2, 4, 5, and 6 (*P* > 0.05). The conclusions of FAS and PPS datasets were consistent (Table 2).

Since the medication had been discontinued by 5 and 6 weeks, many patients were reluctant to come to the hospital, resulting in an increase in missing data, but none of these patients had significant adverse effects and were able to sleep well according to our telephone visits.

Repeated measurement ANOVA of ISI in each treatment stage of the four groups exhibited significant differences in time effect, intergroup effect, and interaction effect (*P* < 0.05). The conclusions of the FAS and PPS datasets were consistent (Table 3).

The ISI scores were compared between the two groups through repeated measurement ANOVA at each treatment stage. Significant differences were observed between Group ZA + *Z*5 and Group Z5, Group ZA + *Z*5 and Group ZA, Group Z5 and Group Z10, and Group Z10 and Group ZA (*P* < 0.05). On the other hand, no significant difference was observed between Group Z5 and Group ZA and Group ZA + *Z*5 and Group Z10 (*P* > 0.05). The conclusions of FAS and PPS datasets were consistent (Table 4).

**Figure 2:**
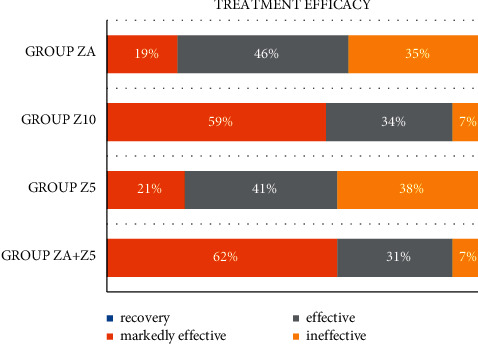
Comparison of the treatment efficacy among the four groups (PPS).

Four weeks after treatment, similar treatment efficacy was observed between Groups ZA + *Z*5 and Z10 (93%) and between Groups Z5 and ZA (62% and 65%, respectively) (Figure 1). Groups ZA + *Z*5 and Z10 exhibited better treatment efficacy than Groups Z5 and ZA (*P* < 0.05). The conclusions of FAS and PPS datasets were consistent. No statistically significant difference was observed between Groups Z5 and ZA (*P* > 0.05) and between Groups ZA + *Z*5 and Z10 (*P* > 0.05). The FAS and PPS datasets were consistent.  Figure 2 shows the treatment efficacy between 4 groups.

### 3.4. Adverse Events

Adverse reactions were observed in 19 participants (Table 5). There were three participants of Group ZA + *Z*5 (9%), six participants of Group Z5 (17%), eight participants of Group Z10 (25%), and two participants of Group ZA (6%). No significant difference was observed in the incidence of adverse events between the groups (Table 6).

## 4. Discussion

More and more evidences show that complementary and alternative medicine (CAM) can supplement other medicine besides mainstream medicine [42–44]. About 12% people taking insomnia have TCM as complement or alternative [36, 37]. TCM is efficacious in insomnia with a mean difference of 3.1 points in Pittsburgh Sleep Quality Index score between TCM and placebo according to a recent systematic review [38].

The treatment efficacy in the four groups is in the order (ZA + *Z*5 = Z10)>(*Z*5 = ZA), indicating that Zaoren Anshen capsule alone is as effective as 5 mg of zolpidem in treating insomnia. The combination of Zaoren Anshen capsule and 5 mg zolpidem tartrate is as effective as 10 mg Zolpidem alone. This combination not only enhances the curative effect of Zaoren Anshen capsule but also reduces the amount of zolpidem needed to treat insomnia, which is beneficial in minimizing the adverse reactions caused by zolpidem.

The incidence rates of adverse events over the 6-week study period in Groups ZA + *Z*5, Z5, Z10, and ZA were 9%, 17%, 25%, and 6%, respectively. The incidence of adverse events in Groups ZA + *Z*5 and ZA was less than 10%, whereas that in Groups Z5 and Z10 was more than 10%. No significant difference was observed in the incidence of adverse events between the groups, which may be due to the small sample size of this study.

A heterogeneity analysis of the 13 studies (1175 patients) indicates that the clinical efficacy of the Zaoren Anshen capsule is superior to that of the conventional hypnotic medicine in treating insomnia; however, the difference is statistically nonsignificant (RR = 1.03; 95% CI = 0.97, 1.09); (*P*=0.34). The adverse reactions caused by Zaoren Anshen capsule treatment are significantly lesser than those caused by conventional hypnotic medicine treatment (RR = 0.20; 95% = (0.14, 0.28), *P* < 0.00001) [41]. Another analysis of 19 studies (1780 patients) indicates that the improvement in sleep quality with Zaoren Anshen capsules is better than that with placebo (RR = −0.90; 95% CI = (−1.56, −0.24); *P*=0.007). Similar to benzodiazepine receptor agonist (RR = 0.17; 95% = (−0.29, 0.64); *P*=0.46), Zaoren Anshen capsules cause lesser incidences of adverse events than benzodiazepine receptor agonists (RR = 0.16, 95% CI = (0.12, 0.23); *P* < 0.001) [40]. The results of clinical effectiveness and adverse reaction incidences in the present study are consistent with those of the aforementioned two meta-analyses, further confirming the effectiveness and safety of Zaoren Anshen capsules in treating insomnia.

An Australian study shows that ZRAS capsule is safe, acceptable, and tolerable, yet not more effective than placebo in the treatment of insomnia [45]. Zaoren Anshen capsules used in this study were manufactured by Australian laboratories. The origin and manufacturing process of the medicinal materials were different from those of Zaoren Anshen capsules made in China. In our study, Zaoren Anshen capsules were taken 5 capsules per night, but in the Australian study, 3 capsules were taken. The difference in drug dosage may lead to the difference in the efficacy.

The strength of this study is its strictly randomized, double-blind control design, and the execution of the study strictly according to the established procedures. The study involves a set of detailed management measures such as random allocation method, allocation scheme hiding, blind application, and completeness of the result data, which avoided subject and researcher bias to ensure that the research results were objective, true, and reliable. The placebo group is not included in this study because of ethical considerations. All the subjects are insomnia patients who suffer from insomnia and seek medical treatment in the hospital. After diagnosis, the patients hope to receive treatment as soon as possible. This study ensures that each patient will receive medical treatment after participating in the study, so as to avoid delaying treatment due to the use of placebo. The side effects of zolpidem are related to its dosage. Therefore, we set up a small dose group and a conventional dose group to observe the difference in the efficacy and side effects.

### 4.1. Limitations

This clinical trial has some limitations. First, the limitation of the study is the short period of intervention and small sample population. This study can be further improved by future research with a larger sample size. Second, the leakage rate during the follow-up of this study was high, mainly because the patients felt that they spent too much time on the way to the hospital. Future research can improve the follow-up process, such as collecting data through telephone or video interview.

## 5. Conclusions

In conclusion, this study showed that, Zaoren Anshen capsule can effectively treat insomnia disorder either alone or in combination with zolpidem tartrate. A preferred combination of TCM Zaoren Anshen capsule with zolpidem can provide a magnified therapeutic efficacy with fewer side effects than zolpidem-only management.

## Figures and Tables

**Table 1:** Participant characteristics by group.

Demographics	ZA + *Z*5 (*N* = 32)	Z5 (*N* = 35)	Z10 (*N* = 32)	ZA (*N* = 32)	Statistics	*P* values
Age (y) mean (SD)	48 (12)	44 (13)	47 (14)	51 (13)	*F* = 1.58	0.20

Height (cm) mean (SD)	164 (8)	162 (7)	163 (8)	163 (7)	*F* = 0.23	0.88

Weight (kg) mean (SD)	58 (9)	56 (8)	59 (11)	61 (9)	*F* = 1.68	0.17

Sex					*χ* ^*2*^ = 1.91	0.59
Male	10	10	14	11		
Female	22	25	18	21		

**Table 2:** Intention-to-treat analysis of ISI scores.

	Mean values (SD)				Between group differences			
	ZA + *Z*5 (*n* = 32)	Z5 (*n* = 32)	Z10 (*n* = 32	ZA (*n* = 32)	FAS (*n* = 131)		PPS (*n* = 98)	
					*F* values	*P* values	*F* values	*P* values
Baseline	14.03 (2.57)	14.09 (4.00)	13.31 (3.82)	14.53 (3.84)	0.62	0.60	0.62	0.60
Week 2	6.80 (2.38)	9.84 (4.57)	6.72 (5.13)	11.11 (3.99)	8.74	<0.001	8.07	<0.001
Week 4	5.34 (2.91)	9.03 (3.78)	5.62 (3.55)	9.04 (4.42)	9.39	<0.001	8.78	<0.001
Week 5	5.34 (2.78)	8.59 (3.97)	5.04 (4.39)	8.24 (3.07)	8.28	<0.001	6.68	<0.001
Week 6	5.14 (3.32)	7.74 (3.83)	4.84 (3.80)	7.15 (2.62)	6.82	<0.001	4.24	0.01

**Table 3:** Repeated measure ANOVA results of ISI scores of the four groups.

Items	FAS (*n* = 131)		PPS (*n* = 98)	
	*F*	*P*	*F*	*P*
The time effect	164.390	<0.001	168.875	<0.001
The intergroup effect	8.267	<0.001	5.560	0.001
The interaction effect	4.083	<0.001	3.960	<0.001

**Table 4:** Repeated measurement ANOVA of ISI scores of the four groups.

Comparison between two groups	FAS	PPS
	*P* values	*P* values
ZA + *Z*5-Z5	0.002	0.013
ZA + *Z*5-Z10	0.719	0.857
ZA + *Z*5-ZA	0.001	0.003
Z5-Z10	0.001	0.010
Z5-ZA	0.665	0.755
Z10-ZA	<0.001	0.002

**Table 5:** Details of the reported adverse events (SAS).

Serial number	Groups	Subject codes	Adverse events	Duration	Severity	Serious adverse events	Turnover	Measures taken for drug test	Corrective treatment	Exit tests	Test drug caused
1	ZA + *Z*5	1032	Dizzy	3 days	Mild	No	Disappear	Continue	No	No	Suspicious
2	ZA + *Z*5	3021	Dry mouth	3 days	Mild	No	Disappear	Continue	No	No	Suspicious
3	ZA + *Z*5	4023	Drowsiness	2 days	Mild	No	Disappear	Continue	No	No	Possible
4	Z5	1013	Constipation and urgency of urination	1 day	Mild	No	Disappear	Continue	No	No	Suspicious
5	Z5	1015	Weak waist and drowsiness	4 days	Mild	No	Disappear	Continue	No	No	Suspicious
6	Z5	1033	Gastrointestinal discomfort	1 day	Mild	No	Disappear	Continue	No	No	Possible
7	Z5	1036	Dry mouth and bitter mouth	2 days	Mild	No	Disappear	Continue	No	No	Possible
8	Z5	1037	Head distension	1 day	Mild	No	Disappear	Continue	No	No	Possible
9	Z5	2025	Drowsiness	3 days	Mild	No	Disappear	Continue	No	No	Possible
10	Z10	1003	Headache	8 hours	Moderate	No	Disappear	Continue	No	No	Possible
11	Z10	1025	Dizzy	4 hours	Mild	No	Disappear	Continue	No	No	Possible
12	Z10	1029	Heart tingling	2 days	Moderate	No	Disappear	Cease	No	No	Impossible
13	Z10	1039	Drowsiness	2 days	Mild	No	Disappear	Continue	No	No	Possible
14	Z10	2012	Drowsiness	2 days	Mild	No	Disappear	Continue	No	No	Possible
15	Z10	2026	Dizzy	1 day	Mild	No	Disappear	Continue	No	No	Possible
16	Z10	3028	Nausea	8 hours	Moderate	No	Disappear	Continue	No	No	Possible
17	Z10	4025	Dizzy	1 day	Mild	No	Disappear	Continue	No	No	Possible
18	ZA	3010	Constipation	2 days	Mild	No	Disappear	Continue	No	No	Suspicious
19	ZA	4007	Mild dull pain in the right upper abdomen	3 days	Mild	No	Disappear	Cease	No	Yes	Impossible

**Table 6:** Incidence of adverse events in the four groups.

Groups	Occurred		Not occurred		Total	*χ2*	*P*
	*n*	Percentage (%)	*n*	Percentage (%)			
ZA + *Z*5	3	9	29	91	32	5.138	0.163
Z5	6	17	29	83	35		
Z10	8	25	24	75	32		
ZA	2	6	30	94	32		
Total	19	15	112	85	131		

## Data Availability

The datasets generated during this study are available from the corresponding author on reasonable request.
